# Testing for Measurement Invariance (MI): Do the Structures of Microaggression, Discrimination, and Resilience Among Black Women Living with HIV Remain the Same Across Time?

**DOI:** 10.1007/s40615-024-02087-w

**Published:** 2024-08-05

**Authors:** Jingxin Liu, Daniel J. Feaster, Naysha Shahid, Kimberly Lazarus, Devina J. Boga, Peyton Willie, Reyanna St. Juste, Maria Fernanda Silva, Layomi Adeojo, Mya Wright, Rachelle Reid, Stephanie Gonzalez, Aarti Madhu, Chelsie Warman, Roxana Bolden, Yue Pan, C. Mindy Nelson, WayWay Hlaing, Allan Rodriguez, Maria L. Alcaide, Gail Ironson, Steven Safren, Ian Wright, Sannisha K. Dale

**Affiliations:** 1 Department of Public Health Sciences, University of Miami Miller School of Medicine, Miami, FL, USA; 2 Department of Psychology, University of Miami, Coral Gables, FL, USA; 3 Department of Economics, University of Miami School of Business, Coral Gables, FL, USA; 4 Department of Medicine (Infectious Diseases), University of Miami Miller School of Medicine, Miami, FL, USA

**Keywords:** Measurement invariance, Latent class analysis, Repeated measure analysis, Black women, HIV, Discrimination, Microaggression, Resilience

## Abstract

Assessing measurement invariance and the interplay of discrimination, microaggressions, and resilience among Black women living with HIV (BWLWH) across time utilizing latent class and repeated measure analysis may provide novel insights. A total of 151 BWLWH in a southeastern U.S. city completed surveys focused on multiple forms of microaggressions and discrimination (race, gender, sexual orientation, or HIV-related) and resilience factors (social support, self-efficacy, post-traumatic growth) at baseline, 3 months, and 6 months. To capture the psychosocial domains of discrimination, microaggressions, and resilience, three latent factors were developed and measured across three time points. Latent class analysis was also conducted to identify and compare meaningful subgroups based on varying levels of discrimination, microaggressions, and resilience reported. Three latent classes were created. MI testing suggested that measurement invariance was partially met (established metric invariance and scalar invariance), and it is possible to compare factor means of discrimination, microaggressions, and resilience across time. Latent factor mean scores of microaggressions and discrimination decreased after 3 and 6 months and increased for resilience after 6 months and varied over time across the three latent classes identified. The subgroup with the lowest level of discrimination and microaggressions and the highest level of resilience reported at baseline, experienced increases in resilience after months 3 and 6. Clinical interventions, research, and policies aimed at promoting resilience and reducing structural and social barriers linked to racism, sexism, HIV stigma, and classism are needed to improve the health and well-being of BWLWH.

## Introduction

HIV has been a consistent challenge to public health, especially among women, who account for more than half of people living with HIV (PLWH) worldwide [1]. In the United States (U.S.), Black women are particularly impacted by HIV, representing nearly 60% of newly diagnosed women living with HIV (WLWH) [2] and having the highest premature mortality rates among PLWH [3] despite making up approximately 14% of the U.S. population. Structural factors such as racism, sexism, and classism often give rise to disparities across the HIV care continuum and impact how Black women navigate day-to-day life on a systematic and interpersonal level. Intersectionality theory — rooted in Black feminist tradition and coined by Kimberlé Crenshaw [4] — highlights how interlocking systems of oppression (e.g., racism, sexism, classism, heterosexism) result in various means of privilege, marginalization, and discrimination [5, 6]. Experiencing mistreatment and prejudice due to the stigmatization of living with HIV is prevalent for BWLWH, and this is heightened by and co-occurs with discrimination and microaggressions as a result of racism and sexism and the intersection of the two (i.e., gendered racism). Due to holding overlapping identities that have been historically marginalized, it is important to understand how experiences of intersectional stigma and discrimination impact BWLWH over time. Furthermore, the role of resilience — the ability to bounce back from or in the face of adversity — in response to stressful life experiences rooted in intersectional stigma across time is relevant and necessary in identifying protective factors that may improve the health and well-being of BWLWH.

Racial, gender, and HIV-related discrimination, as well as gendered racial microaggressions (GRM) — daily insults, passive-aggressive behaviors, and environmental slights that function at the intersection of racism and sexism [7] — have been identified as prominent stressors among BWLWH that have been linked to negative consequences at the institutional and interpersonal level [8]. For instance, higher levels of HIV-related discrimination, racial discrimination, and GRM have been linked to more barriers to care such as difficulty accessing medical facilities, lack of financial resources, and community stigma [8]. Furthermore, discrimination and microaggressions stemming from racism, sexism, and HIV stigma have also been shown to have an adverse impact on mental health among BWLWH [9-11]. Several studies have found that HIV-related discrimination and stigma, racial discrimination, and gendered-racial microaggressions have been significantly associated with depression and trauma symptoms among BWLWH [9-11]. In addition to mental health, experiences of discrimination have been connected to HIV-related outcomes among other groups disproportionately affected by HIV. Previous studies primarily conducted among Black men [12] or men who have sex with men [13] living with HIV, reported that discrimination related to race, HIV status, and sexual orientation negatively impacted medication adherence, engagement in care, and HIV viral suppression [12, 13]. Additionally, among Black women who have sex with women, discrimination rooted in heterosexism, and racism was related to lower engagement in care [14]. Such findings highlight the adverse impact intersectional stigma and discrimination have on the mental and physical health of PWLH and individuals who hold multiple marginalized identities.

Despite being disproportionately exposed to stressful life experiences due to various isms, people navigating the world with intersectional stigmatized identities often build and rely on resilience, which represents the ability to cope and adapt when faced with adversities and inequities [15]. For PLWH, which disproportionately affects minoritized and marginalized populations, resilience may be beneficial for unique reasons. Depression and anxiety are two of the most pervasive mental health disorders among PLWH [16]. These mental health issues are often associated with low treatment adherence and exposure to discrimination and stigma [16]. Among PLWH, higher levels of resilience resources have been associated with lower levels of depression and anxiety [17], better quality of life, and reduced HIV severity (e.g., lower HIV viral load, higher CD4 T cell counts) [16, 18]. Moreover, studies also have shown relationships between resilience, microaggression, and discrimination [19-21]. Among a sample of BWLWH, a novel approach utilizing latent class analysis found that women who experienced lower levels of adversity (trauma, discrimination, microaggressions) and higher levels of resilience resources tend to have lower PTSD or depressive symptoms [22]. This was the first and only study [22] to utilize this statistical method to explore the interplay of intersectional microaggressions, discrimination, resilience, and mental health among BWLWH; however, this study was cross-sectional. Therefore, additional investigations examining how varying levels of discrimination, microaggressions, and resilience may influence the health and well-being of BWLWH are warranted. To achieve this long-term goal, one important step is to understand how these psychosocial factors interact with each other over time. The current study aims to capture the unique interplay of intersectional discrimination/microaggressions and resilience, and how these psychosocial factors may fluctuate across time through utilizing longitudinal repeated data and latent classes of discrimination, microaggression, and resilience factors among BWLWH.

## Methods

Data for the current study was obtained between October 2019 and April 2021 from the Monitoring Microaggressions and Adversities to Generate Interventions for Change (MMAGIC) project, which is a longitudinal study of BWLWH in a metropolitan area in the Southeastern United States. IRB approval was obtained, study objectives were fully explained to participants, and written informed consent was obtained. Recruitment efforts were described in a previous publication [23]. In sum, participants who previously gave consent to be contacted for other research programs were contacted, and flyers and posters were distributed at places where BWLWH might frequently appear, such as the following: health centers, hospitals, community events, and the facilities of community-based organizations.

A phone screening was conducted by study staff for individuals who expressed interest and the inclusion and exclusion criteria were applied to determine their eligibility. The specific inclusion criteria of participants were as follows: (1) living with HIV, (2) identify as Black and/or African American, (3) age 18 or older, (4) Cis-gender woman, (5) English speaking, (6) capable of completing and fully understanding the informed consent process and procedures, and (7) own a cell phone with text and internet capacity [24]. Participants were excluded if they (1) did not meet the above-mentioned criteria, (2) were unable to completely understand the consent process and study procedures, or (3) had significantly interfering mental health symptoms such as active psychosis. A total number of 151 participants were included in the current study.

A sociodemographic survey was distributed at baseline and gathered information on the participants’ age, ethnicity, education, sexual orientation, annual income, housing status, employment, and relationship status. Measures of microaggression, discrimination, and resilience were administered to participants at each study visit to capture psychosocial factors. Data used herein include repeated assessments at baseline and 3 months and 6 months after baseline.

### Latent Factors

These factors were assumed to cause the observed psychosocial data collected. Each indicator was the established scale score of a validated instrument, rather than the individual items of these instruments. In this research, three latent factors were defined for three separate conceptual domains.

#### Microaggression Latent Factor

**Fig. 1:**
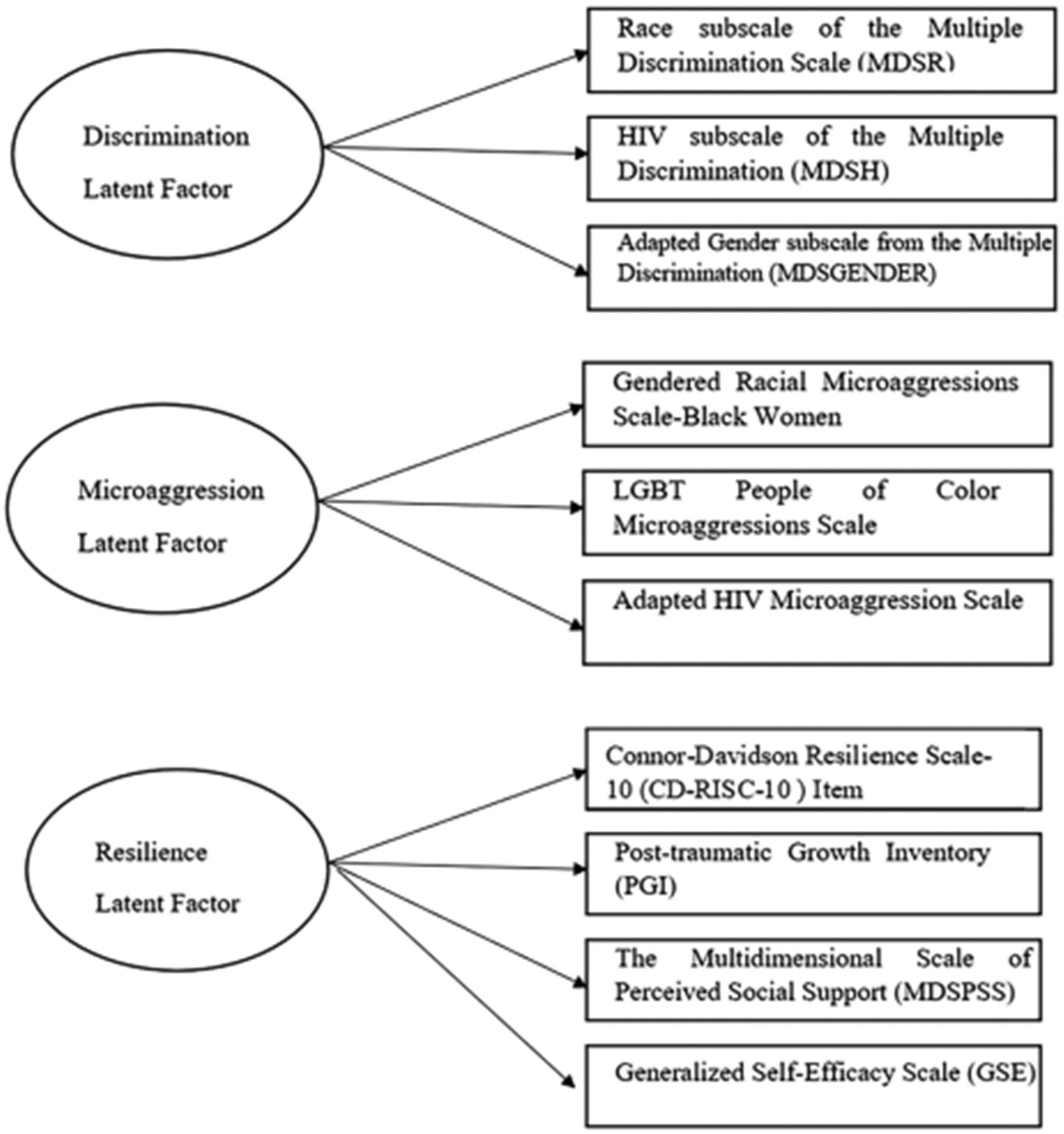
Constructs of latent factors with observed indicators

This factor was measured by three scales (Fig. 1): the *Gendered Racial Microaggressions Scale-Black Women* [7] which measures gendered racial microaggressions (microinvalidations, microinsults, and microassaults) experienced by women on the basis of them being both Black and Women, the *LGBT People of Color Microaggressions Scale* [25] which assesses microaggressions experienced by racially/ethnically minoritized LGBT adults, and the *HIV Microaggression Scale* [7, 25] which captures an individual’s experiences of insults on the basis of them living with HIV.

##### Gendered Racial Microaggressions Scale for Black Women

The GRMS-BW is a 26-item scale that measures the frequency (how often each event happened) and stress appraisal (how stressful each experience was) of gendered racial microaggressions experienced by Black women. Sample items include “Someone has made me feel unattractive because I am a Black woman” and “I have been told I am too independent.” Participants were requested to select how often the event happened to them on a scale of 0–5 (0, never; 1, less than once a year; 2, a few times a year; 3, about once a month, 4, a few times a month; 5, once a week or more). The total score was calculated for each participant and could range from 0 to 130; higher scores indicated more frequent experiences of gendered racial microaggressions [7]. Literature has shown the GRMS-BW to have good validity and reliability.

##### The Lesbian, Gay, Bisexual, and Transgender (LGBT) People of Color Microaggressions Scale (LGBT-PCMS)

This 18-item self-report measure has demonstrated good validity and reliability (*α* = 0.95) [25] in prior research. The LGBT-PCMS assesses the unique types of microaggressions experienced by LGBT racially/ethnically minoritized adults. Individuals were asked to indicate how frequently (never, less than once a year, a few times a year, about once a month, a few times a month, once a week, or more) an event has happened to them in their lifetime. A total score was calculated for this scale and could range from 0 to 90. A higher score indicates higher levels of microaggressions experienced by LGBT People of Color.

##### HIV Microaggression Scale

A 14-item self-report measure was used to capture an individual’s experiences of insults on the basis of the individual living with HIV. Sample questions include “You heard someone say, ‘I’m HIV negative, I’m clean’” and “Someone assumed you don’t or shouldn’t have sex because of your HIV status.” Participants were required to select how often in the past 6 months they experienced such an event on a scale of 0–5 (0, never; 1, rarely; 2, sometimes; 3, often; 4, not applicable; and 5, choose not to answer). A total score was calculated for each participant and could range from 0 to 42. Higher scores suggested a higher frequency of experiences with HIV microaggressions. This scale has shown good validity (*α* = 0.90) and reliability [26] in previous literature [26].

#### Discrimination Latent Factor

This factor was measured by three subscales (Fig. 1): the Race subscale of the Multiple Discrimination Scale (MDSR) [13] (13 items), HIV subscale of the Multiple Discrimination Scale(MDSH) [13] (13 items), and Adapted Gender subscale from the Multiple Discrimination Scale (MDSGENDER) [13] (13 items). The score of each domain was calculated for each participant and could range from 0 to 13. Higher scores indicated higher levels of discrimination experience.

##### Race Subscale of the Multiple Discrimination Scale (MDSR)

The Multiple Discrimination Scale’s Race subscale consists of 13 items. Participants were requested to answer yes or no (yes, 1; no, 0) to whether they had experienced any of the discriminatory events specified based on race. Sample questions include “In the past year, were you denied a place to live or did you lose a place to live because you are Black or African American?”. A total score was calculated for each participant and could range from 0 to 13. Higher scores indicated higher levels of experience of race-related discrimination. In prior literature, the MDS has shown good validity and reliability (*α* = 0.85) [13, 27, 28].

##### HIV Subscale of the Multiple Discrimination Scale (MDSH)

This 13-item subscale captures experiences of HIV discrimination. Sample questions include “In the past year, were you denied a place to live or did you lose a place to live because you are HIV positive?”. Participants were requested to answer yes or no (yes, 1; no, 0). A total score was calculated for each participant and could range from 0 to 13. Higher scores pointed to higher levels of experiences of HIV-related discrimination. The MDSH has shown good validity and reliability (*α* = 0.85) in previous literature [13, 27, 28].

##### Adapted Gender Subscale from the Multiple Discrimination Scale (MDSGENDER)

To capture gender discrimination, 13 items were adapted from the original Multiple Discrimination Scale (MDS) [25]. An example of an item includes “In the past year, were you denied a job or did you lose a job because of your gender?”. Participants were requested to answer yes or no (yes, 1; no, 0). A total score was calculated for each participant and could range from 0 to 13. Higher scores suggested higher levels of experience of gender-related discrimination. In prior studies, the original MDS has demonstrated good evidence of validity and reliability (*α* = 0.88) [25].

#### Resilience Latent Factor

This factor captures resilience at the individuals and interpersonal level and was measured by four scales (Fig. 1): the Connor-Davidson Resilience Scale-10 Item (CD-RISC-10) [15] (10 items) to capture resilient traits and coping, Post-traumatic Growth Inventory (PGI) [29] (21 items) to capture psychosocial growth following a traumatic experience, the Multidimensional Scale of Perceived Social Support (MDSPSS) [30] (12 items) to measure levels of social supports from family, friends and significant others, and Generalized Self-Efficacy Scale (GSE) [31] (10 items) to assess individual’s belief that they can cope with adversities.

##### Connor-Davidson Resilience Scale-10 Item (CD-RISC-10)

This ten-item scale measures resilient coping and traits. The CD-RISC-10 is a widely used measure with evidence of good validity and reliability (*α* = 0.92) [15]. Sample questions include “I am able to adapt when changes occur” and “I try to see the humorous side of things when I am faced with problems.” Answer choices ranged from 0 to 4 (0, not true at all; 1, really true; 2, sometimes true; 3, often true; 4, true nearly all the time). A total score was calculated for each participant and could range from 0 to 40. Higher scores reflect a greater ability to adapt or greater resilience.

##### Post-traumatic Growth Inventory (PGI)

The Post-traumatic Growth Inventory is a 21-item scale assessing psychological growth following a traumatic event. Sample items include “My priorities about what is important in life” and “I discovered that I’m stronger than I thought I was.” Response options range from 0 to 5 (0, I did not experience this change; 1, very small degree; 2, small degree; 3, moderate degree; 4, great degree; 5, very great degree). A total score was calculated for each participant and could range from 0 to 105. Higher scores suggested greater ability to respond to changes that have occurred in life. The PGI has shown good internal consistency reliability in prior studies.

##### The Multidimensional Scale of Perceived Social Support (MDSPSS)

This 12-item scale captures levels of emotional support. Participants were asked to respond to statements such as “There is a special person who is around when I am in need”, on a scale of 1–7 (1, very strongly disagree; 2, strongly disagree; 3, mildly disagree; 4, neutral; 5, mildly agree; 6, strongly agree; 7, very strongly agree). A total score was calculated for each participant and could range from 0 to 84. Higher scores indicated a better experience of receiving perceived social support. Previous literature has demonstrated this scale to have good validity and reliability (*α* = 0.94) [32].

##### Generalized Self-Efficacy Scale (GSE)

The General Self-Efficacy is a widely used scale that has shown great validity and reliability in prior research [31]. The ten-item scale consists of questions such as “I can always manage to solve difficult problems if I try hard enough”. Answer choices range from 1 to 4 (1, not true at all; 2, hardly true; 3, moderately true; 4, exactly true). A total score was calculated for each participant and could range from 0 to 40. Higher scores reflected a greater ability to self-manage difficulties.

Figure 1 visually displays three latent factors of discrimination, microaggression, and resilience and their corresponding observed measures. These latent factors are estimated at the three time periods. It is observed that measurements of MDSPSS at time 1 (3 months after baseline) and time 2 (6 months after baseline) were highly correlated; thus, values of MDSPSS at time 1 were used in the indicator for time 2 in this study.

## Establishing Measurement Invariance Across Time

Previous studies have reported tests of equivalent variance–covariance matrices [33, 34] to establish measurement invariance; however, this approach has been thought to be overly restrictive since the overall test may mask small but important differences [35]. Therefore, this study proposed a series of measurement models with increasing constraints on parameters across times of assessment. If the null hypothesis was rejected on one of the steps in this series, the observed indicators and applied restrictions were examined to understand the parameters that contributed most to the rejection [36]. Then, in a reanalysis of this step, parameters that contributed most to the rejection were set free in the next measurement model, and this process continued until the null hypothesis of a less restrictive model was not rejected. This procedure allows for partial invariance across different parts of the variance–covariance matrix of the indicators.

The difference in the Comparative Fit Index (CFI) and Root Mean Square Error of Approximation (RMSEA) [37] between a more restricted model and a baseline model was calculated. Studies have also reported the likelihood ratio test to detect the improvement between models; however, these tests are frequently overpowered [36]. In this study, a small change in fit indices (less than 0.01 on the confirmatory fit index) was expected to meet the criteria of measurement invariance [37-39]. Specifically, three phases of measurement invariance tests were performed: (1) configural invariance, (2) metric invariance, and (3) scalar invariance.

Configural invariance allows the examination of the appropriateness of the hypothesized indicators across the factor being examined (herein the factor is time), which is also called the baseline model (Step 1). Configural invariance implies that the same set of items measures the same underlying factors across time. This study specified all the relationships between items of psychosocial domains (microaggression, discrimination, and resilience) without constraints. Three latent factors at each visit were created. As an example, latent microaggression at visit time 1 or 2 or 3 was built on indicators of Gendered Racial Microaggressions Scale-Black Women (GRMS), LGBT People of Color Microaggressions Scale (LGBT), and HIV Microaggression Scale (HIVM) at the corresponding time. The latent factor for microaggression at baseline was created with baseline manifested variables of GRMS, LGBT, and HIVM, the latent factor for microaggression at time 2 was created by indicators obtained from time 2, and the latent factor of microaggression at time 3 was created by indicators from time 3. Full correlations of repeated measures were also specified, including correlations between GRMS1, GRMS2, and GRMS3. This same logic was applied to the formation of latent factors of discrimination and resilience.

Factor loadings were constrained further to test metric invariance [33] also known as weak invariance [40]. Metric invariance implies that a unit increase on a particular item at one time period is the same proportionate increase in the level of the latent construct at another time period, and the coefficient of proportionality was represented by the estimated loading. Particularly, factor loadings of LGBT and HIVM on microaggression, MDSH and MDSGENDER on discrimination, and the factor loadings of PGI, MDSPSS, and GSE on resilience were fixed over time (Step 2).

To compare levels of factors across time, the zeropoint on the underlying latent factors need to be taken into consideration, which required additional restrictions on intercepts (the means of indicators when latent factors were zero). This is referred to as scalar invariance [33] or strong invariance [40]. To test the scalar invariance, intercepts were constrained building on the results of the final metric invariance measurement models [33]. If the null hypothesis fails to be rejected, then it implies equal item intercepts or no difference in normative levels of the item across time. Model fit indices included the value of Akaike (AIC), Bayesian (BIC), sample-size adjusted BIC, RMSEA, CFI, and TLI [38].

The final part involves the establishment of strict equivalence [40], which contains two sets of tests in addition to both metric and scalar invariance. Specifically, those are the tests of equal item variances across time, and equal factor variances over time. The failure to establish equal item variances and/or factor variances over time leads to the situation that standardized loadings and factor reliability vary across time even though metric invariance holds [36].

### Factor Means Over Time

This study aimed to compare the means of latent factors of microaggression, discrimination, and resilience at three visits to understand how those psychosocial factors fluctuate over time. Establishing measurement invariance (MI) of latent constructs is a precondition [41] for the meaningful comparison of latent factors [42] of microaggression, discrimination, and resilience over time. This step ensures the measurement structures of the latent factors and survey items are stable over time. Though testing MI is methodologically challenging, this research proposed to conduct this analysis through establishing equivalence of factor loadings, intercepts, and residual variances across time [43].

### Prediction of Factor Scores

Corresponding baseline indicators of three psychosocial measurements were utilized to form factor scores of microaggression, discrimination, and resilience. Latent factors were estimated as the maximum of the posterior distribution of observed items [44, 45].

### Latent Class Analysis

To gain insights into differences in latent constructed domains among BWLWH, we further divided the study sample into subgroups using latent class analysis (LCA) using baseline data. This analysis further examined how the values of observed psychosocial indicators as well as three latent factor scores differed over the three time periods between latent classes.

LCA is used to discover hidden groups from an observed set of continuous indicators [46, 47], which is a person-oriented approach [48, 49]. To identify the number of profiles among 151 BWLWH, an iterative modeling process was performed using Mplus 8.4. To be specific, a series of models was performed with varying numbers of profiles; fit indices of each model were collected and compared against each other to select an optimal number of latent classes [50]. The fit indices include the Bayesian information criterion (BIC) [51, 52], the Akaike information criterion (AIC), sample-size adjusted Bayesian information criterion (SABIC), and entropy. The BIC and AIC reward parsimony in a model; thus, lower BIC and AIC values represent a better fit [50, 53]. An entropy value of 0.80 or higher indicates good profile classification of individuals [54] with minimal uncertainty [53, 55]. BIC has been shown to be the most reliable indicator [56] in judging the appropriate number of latent groups, but other reliable indicators include entropy, AIC, and SABIC.

Baseline variables of microaggression, discrimination, and resilience were selected as indicator variables. The completeness of these indicators ensured adequate sample sizes to obtain sufficient statistical power to identify meaningful subgroups [57]. A small sample size usually limits the potential to detect meaningful differences between latent classes for observed indicator variables; thus, enough sample enables sufficient statistical power to identify meaningful subgroups [57]. In total, ten continuous indicator variables (Table 3) were included to generate latent class models. To identify the appropriate number of profiles, a sequence of models varying the number of profiles was estimated [58].

### Automatic BCH Approaches

It was also of interest to explore relations between these baseline latent categorical variables and auxiliary observed variables (continuous), including age, income, and years since diagnosis as well as the factor means from the invariance analysis over time. To achieve this purpose, the automatic BCH approach was applied, which overcame the disadvantage of a one-step approach that may alter latent classification information if both the latent class model and latent regression model are combined in a single model [43] and also outperforms the three-step approach by avoiding shifts in latent class in the final stage [59].

First, a regular latent class analysis (LCA) with latent class indicators variables only was conducted. Next, the most likely class variables were located by computing the latent class posterior distribution that was obtained from the estimated LCA model developed during the previous step. In Mplus, this variable was automatically created using the SAVEDATA command with the option SAVE = CPROB [59]. In the third step, the most likely class variable was regarded as a latent class indicator variable and was regressed on predictor variables. In Mplus, we used the AUXILIARY option in the VARIABLE command to produce the BCH estimation of the distal outcome model and the latent class predictor model [59].

## Results

The study population consisted of 151 BWLWH with an average age of 53 years old, 96.7% of whom were non-His-panic, 64% of whom had received high school education and above, and 68.2% of whom were living on disability (Table 1). Across time, the sample retention rate was high (T2 98%, T3 95%), although there was some slight decrease in the study sample from T1 to T3 (T1 *n* = 151, T2 *n* = 148, T3 *n* = 144).

### Overview Measurement Invariance

Table 2 illustrates fit indices of the series of measurement models with increasing constraints on parameters across times of assessment. A lower AIC or BIC value, a lower RMSEA, and a larger CFI indicate a better fit. We used the CFI and RMSEA to assess whether the final models improved or did not sufficiently degrade the fit compared to the initial model under all types of invariance tests. We will describe each step in this process, below.

#### Configural Invariance

The initial model constrained one-factor loading of each latent construct (microaggression, discrimination, resilience) to one to identify latent factors. Specifically, factor loadings of the Gendered Racial Microaggressions Scale (GRMS), Race subscale of the Multiple Discrimination Scale (MDSR), and Generalized Self-Efficacy Scale (GSE) at each visit time were set to one. In addition, the factor means of latent constructs were zero, and intercepts were free to vary across time. This initial model had a CFI of 0.907 and RMSEA of 0.058. Standardized loadings for all indicators at all times were sufficient to indicate configural invariance except for the LGBT People of Color Microaggressions Scale (LGBT-PMS) at the 6-month model of microaggressions, the Multidimensional Scale of Perceived Social Support (MDSPSS) in the resilience factor at baseline, and GSE within resilience at time 2. All indicators were retained in later steps so that the indicator could contribute to the latent construct at all times.

#### Metric Invariance

Next, all the factor loadings were set to be equal across time to test metric invariance. This model had a CFI of 0.865 and an RMSEA of 0.068. To further improve the model fit under the test of metric invariance, three specific factor loadings were set free: LGBT in the microaggression factor at time three, GSE within resilience at time two, and GSE at time three on resilience at time three. Since GSE was not measured at time point two, the values of GSE at time one were retained in time two. This improved model had a CFI of 0.914 and an RMSEA of 0.054. This model did not differ much from the model in which loadings were unconstrained (∆RMSEA = 0.004 < 0.01 and the CFI was improved) and indicated partial metric invariance. This metric invariance implied that a unit increase in one manifested item implies an equal difference in the latent construct across all-time visits. The actual loadings for each of the indicators at this step can be seen by examining the columns under metric invariance in Table 3.

#### Scalar Invariance

The following model constrained the item intercepts to be equal across time to test scalar invariance. This initial model of scalar invariance had a CFI of 0.852 and RMSEA of 0.070; however, this model showed a poorer fit to the data than the model with equal loadings and free intercepts. To improve the model fit for testing scalar invariance, several intercepts were allowed to vary across times. Specifically, the following intercepts were allowed to vary at the time indicated from the other times: Gendered Racial Microaggressions Scale (GRMS) at baseline, HIV subscale of the Multiple Discrimination (MDSH) at baseline, Adapted Gender subscale from the Multiple Discrimination (MDSGE) at baseline, LGBT People of Color Microaggressions Scale (LGBT-PCMS) at time 3, and Connor-Davidson Resilience Scale-10 Item (CD-RISC-10) at 6 months. This implies that the value of those indicators associated with a zero score on the associated latent variable differed at that particular time than at other times. Because there are indicators in each latent factor that were invariant across all times, comparisons of means of the latent factors are possible. This final model within the scalar invariance step had a CFI of 0.906 and an RMSEA of 0.056. The actual estimated intercepts for these two models can be seen by examining the columns under Scalar Invariance in Table 4.

#### Strict Equivalence

The first step in accessing strict equivalence is to test for equal residual variance of the items (each indicator variable) across time. The initial model imposing this constraint had a CFI of 0.773 and an RMSEA of 0.085. To find a better fitting model that might support partial invariance of item variances, the following residual variances were freed at the time indicated: Post-traumatic Growth Inventory (PGI) at time 1, Adapted Gender subscale from the Multiple Discrimination (MDSGENDER) at time 1, LGBT-PCMS at time 3, and Race subscale of the Multiple Discrimination Scale (MDSR) at time 3 were freed up. The modified model was improved with a higher CFI of 0.910 and a lower RMSEA of 0.054. This indicated that most of the residual item variances were invariant across time. The actual variance and residual variance for each of the indicators at this step can be seen by examining the columns under metric invariance in Table 5.

The second step of the test for strict equivalence is to constrain factor variances to be equal over time. The model for this test had a CFI of 0.89 and RMSEA of 0.06. A second model was estimated which allowed the variance for the discrimination latent factor to vary at all times. This model had a CFI of 0.904 and RMSEA of 0.055. Compared to the initial configural model, this model had an improved (lower) RMSEA but the change in CFI of – 0.003 was not consequential which indicates that the series of restrictions did not change the fit of the measurement model. For factor means of the final model estimates are as follows: latent factor of microaggression (MICRO) T2 (estimate [Est] – 0.494, standard error [SE] 0.101, *Z*-statistics (*Z*) – 4.886, *P*-value (*P*) 0), MICRO T3 (Est – 0.662, SE 0.113, *Z*-5.831, *P* 0), latent factor of discrimination (MACRO) T2 (Est – 1.481, SE 0.207, *Z* – 7.146, *P* 0), MACRO T3 (Est – 1.758, SE 0.226, Z – 7.789, *P* 0), latent factor of resilience (RESIL) T2 (Est 0.144, SE 0.609, Z 0.236, *P* 0.814), and RESIL T3 (Est 2.815, SE 0.744, Z 3.784, *P* 0). The standardized coefficients are illustrated in Table 6. The standardized coefficients showed good reliability in measurements of latent factors of microaggression at times 1 and 2. However, the LGBT-PCMS measurement did not show good reliability in measuring the latent factor of microaggression at time 3. The reliability of the discrimination latent factor was good across three time points, as well as the resilience latent factor at both times 1 and 3 (Table 6).

### Summary of Measurement Invariance

None of the steps of measurement invariance showed full invariance. At each step (Table 2), some of the restrictions were relaxed. Comparing factor means across time is possible because at least some (and in reality, most) of the indicator items showed metric invariance and scalar invariance. Metric invariance means that a unit increase in the indicator variable maps to the same unit increase in the latent factor at each time. Scalar invariance means that the same value of the indicator item is associated with the zero point of the latent factor at all times. Strict invariance is not necessary to make meaningful comparisons across time. This type of invariance does help statistical power slightly because it implies a test of the difference of means with equal variances. Also, because the metric invariance step only constrains the raw factor loading and the standardized loading of an item indicator also involves the residual variance of that indicator and the variance of the latent factor, strict invariance over time is required to have equal standardized loadings over time.

### Means of Latent Factors Over Time

**Fig. 2:**
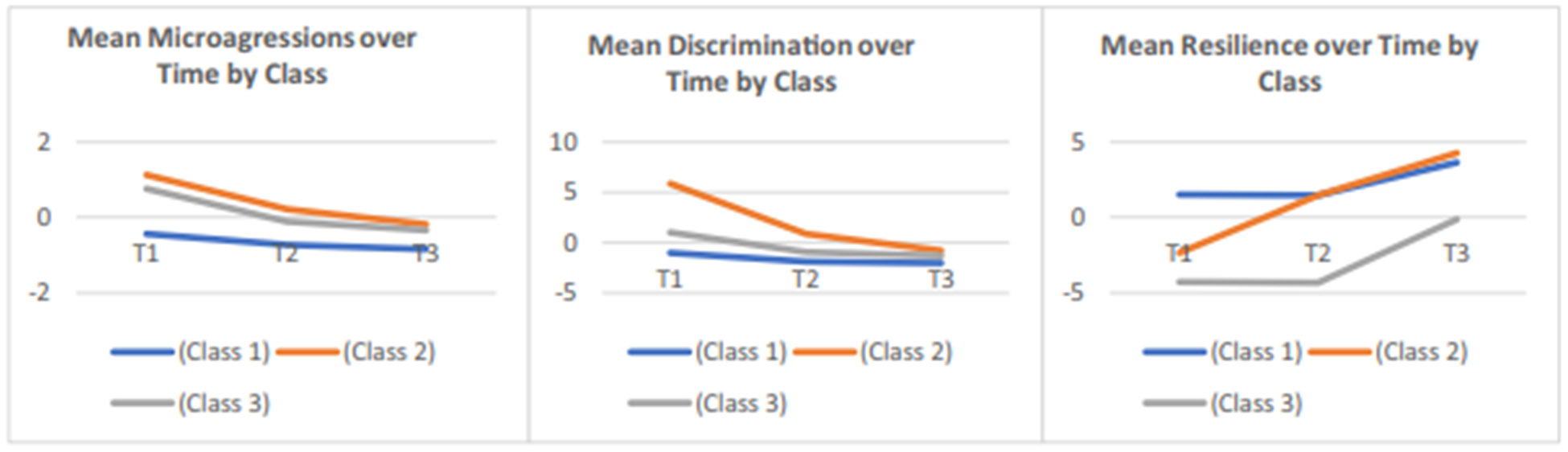
Mean scores of microaggressions, discrimination, and resilience over time by class

Factor means of microaggression, discrimination, and resilience at baseline were set to zero for identification and therefore are not shown in the model results. The means of latent factors over time showed distance away from the value of baseline (set as 0). As seen in Fig. 2, factor means of microaggression, discrimination, and resilience at later times are significantly different from zero and therefore have changed significantly from baseline except resilience latent factor at time 2, though results suggested a trend of improvement since baseline. Specifically, means of microaggression and discrimination latent factors decreased at both 3- and 6-month intervals, while resilience factor mean increased at 6-month interval.

## Latent Class Analysis (LCA)

The indicators of microaggression, discrimination, and resilience at baseline were utilized to conduct latent class analysis. A series of models with 2–5 profile solutions was conducted. Values of AIC, BIC, and SABIC declined as the number of latent classes increased: 2 classes (AIC 9360.994, BIC 9454.530, SABIC 9356.418), 3 classes (AIC 9183.843, BIC 9310.569, SABIC 9177.643), 4 classes (AIC 9111.320, BIC 9271.236, SABIC 9103.496), and 5 classes (AIC 9052.873, BIC 9245.979; SABIC 9043.425). However, the value of entropy varied (two classes 0.944, three classes 0.920, 4 classes 0.860, and five classes 0.875). The two-class solution and three-class solution had an entropy over 0.9, which indicates good separation within the classification of persons. In addition, the AIC and BIC were lower for the three-class solution relative to the two-class solution.

**Fig. 3:**
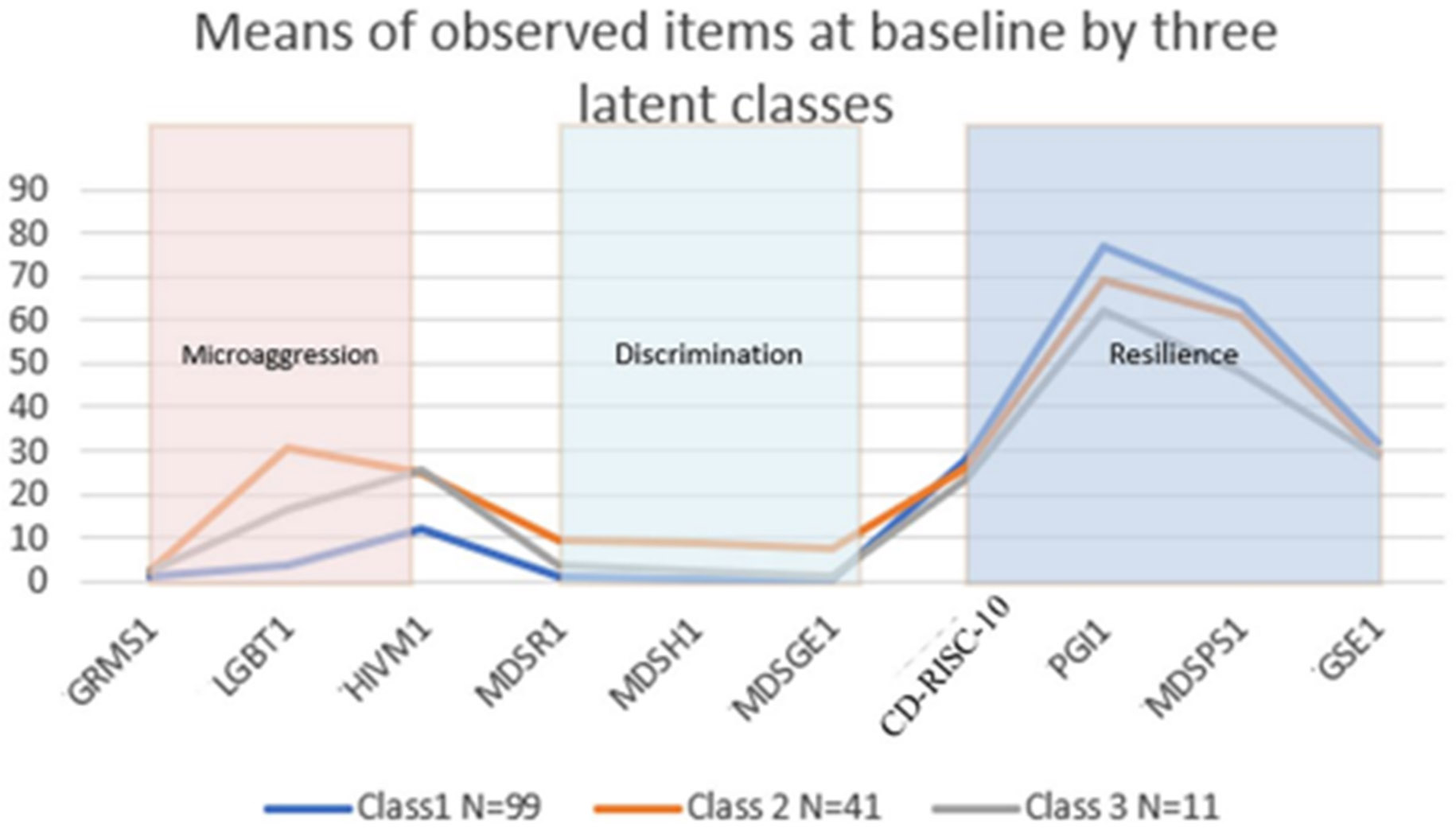
Means of observed items at baseline by three latent Classes GRMS: Gendered Racial Microaggrcssions Scale-Black Women. LGBT: LGBT People of Color Microaggressions Scale. HIVM: Adapted HIV Microageression Scale. MDSR: Race subscale of the Multiple Discrimination Scale. MDSH: HIV subscale of the Multiple Discrimination. MDSGE: Adapted Gender subscale from the Multiple Discrimination. CD-RISC-10: Connor-Davidson Resilience Scale-10 Item. PGI: Post-traumatic Growth Inventory. MDSPSS: Multidimensional Scale of Perceived Social Support. GSE: Generalized Self-Efficacy Scale.

After the overall consideration of values of AIC, BIC, SABIC, and entropy, as well as the visual plot of latent classes by indicator variables (Fig. 3), the model with the three-class solution was selected as the model with the best classification. The sizes of the three subgroups were 99, 41, and 11.

The mean scores of observed items vary across latent groups. Specifically, the first class (*N* = 99; lMlDhR: low microaggression, low discrimination, high resilience) had the lowest values of all indicators of latent domains of microaggression (GRMS, LGBT-PCMS, HIVM) and discrimination (MDSRACE, MDSHIV, MDSGENDER) and had the highest values of indicators of resilience factor (CD-RISC-10, PGI, MDSPS, GSE). This suggested that this subclass was the group with the fewest experiences of microaggression and discrimination and the highest resilience. The second class (*N* = 41; hMhDmR: high microaggression, high discrimination, moderate resilience) had higher LGBT-PCMS value of microaggression factor and higher values of items of discrimination and resilience factors compared to the last class (*N* = 11). The third class (*N* = 11; mMmDlR) had moderate microaggression, moderate discrimination, and the lowest resilience among the three classes. This suggested this subgroup experienced the most microaggression and discrimination among all three subclasses and ranked second in terms of resilience. Such conclusions can also be visually seen in Fig. 3.

The examination of the factor means over time within the baseline latent classes showed that when taking class 1 (*N* = 99) as the reference group, latent class 2 (*N* = 41) had higher factor scores of microaggression at baseline, and higher discrimination at baseline and last visit, and resilience at first two visits, and class 3 (*N* = 11) had higher factor scores of microaggression at baseline and last visit time, higher discrimination at visit time 2, and higher resilience scores at last two visits. When comparing latent class 3 with class 2, the former group had lower microaggression at time 2, higher discrimination at time 2, and lower resilience at baseline (Table 7).

Lastly, factor correlations between the latent factors (at T1, t2, T3) indicated that the latent factors are correlated with each other (see Supplementary Table 12). In addition, the sample size for response across each measure that contributed to factors decreased somewhat over time (see Supplementary Table 13). With a baseline study sample of 151, sample size for measure response ranged from 144 to 151 at baseline, 136 to 146 at T2, and 128 to 135 at T3.

## Discussion

In this study, we addressed various objectives using both variable-centered and person-centered approaches to enhance our understanding of multiple discriminations and microaggressions faced by Black women living with HIV in addition to displays of resilience to counter intersectional discrimination and stigma. Previous research has highlighted how the interplay and variability in the experiences of intersectional adversity and resilience impact health outcomes in BWLWH differently; however, these findings were limited to cross-sectional data analysis. [22] The current study sought to build upon these findings with longitudinal data and dive deeper to examine means of latent factors of microaggression, discrimination, and resilience at three visits. The establishment of measurement invariance (MI) is a pre-requirement. First, this study employed measurement invariance methods to assess the validity of estimating and comparing three-factor mean scores for microaggressions, discrimination, and resilience based on various indicators related to each latent variable. Next, this study examined the changes in each latent factor over three time points and finally used a person-centered approach of latent class analysis to characterize the sample of BWLWH to understand the patterns of microaggression, discrimination, and resilience experienced by subgroups over the course of three time points. Taken together, these results provide important insights into the underlying psychosocial characteristics and meaningful comparisons of how these experiences exist and change over time. Thereby providing a good foundation for future inquiries into the relationship between intersectional adversities and other factors of interest among people living with HIV (such as HIV viral suppression, a key goal of HIV treatment).

In order to accurately make comparisons between microaggression, discrimination, and resilience experiences between the three time points measurement invariance testing was necessary. The indicators included in the development of the latent factors in this study were crucial to authentically study intersectionality. As previously mentioned in the literature, discrimination and microaggressions are differentially experienced in relation to race, gender, sexual orientation, and HIV status. To best mirror what is found in the literature and lived experiences, our microaggression, discrimination, and resilience latent factors reflected multiple levels and sources measured through the indicators. The findings of the three levels of measurement invariance testing (configural, metric, and scalar) implied that partial measurement invariance was met. This indicated that it would be statistically appropriate to make comparisons between the factors means across time. In testing our configural invariance, results suggested a similar factor structure over three time visits. In our metric and scalar invariance testing, at least some (and in reality, most) of the indicator items showed invariance. Specifically, the establishment of metric invariance indicated that a change in items at a one-time point reflected a proportionate change of the latent construct at a later time. Due to the fact that some item loadings and item intercepts were not invariant over time, factor scores that accounted for this invariance were created to facilitate comparisons in subsequent analysis. In our strict equivalence testing, we found that the indicator related to microaggression experiences for sexual minorities (LGBT-PCMS) did not show good reliability in measuring the latent factor of microaggression at time point 3. This could be possibly explained due to the small sample size of individuals that answered these questions (about 12% of the sample). Previous literature [25] has also found that scores were higher for men and those of Asian race which may indicate further assessment for the use of this tool among Black women.

MI testing suggested that measurement invariance was partially met (established metric invariance and scalar invariance), and it is possible to compare factor means across time. Thus, after establishing the appropriateness of factor mean comparisons, we found in the comparison across time with the baseline as the reference group that factor means were significantly different from zero at the 3- and 6-month time points. This study found that microaggression and discrimination factor means decreased after 3 months and 6 months, and for resilience, there was an increase after 6 months. Resilience at time point 2 was trending at an increase; however, this increase was not statistically significantly different from baseline. These findings may help us to understand the natural progression of experiences as well as the impacts of the environment on our sample. As the parent study was not delivering an intervention, the microaggression, discrimination, and resilience score data found in this study may serve as a reference or baseline for future intervention work allowing us to see the true comparisons trending over time. These findings also encourage further research to understand the relationship between intersectional adversities and resilience. Ensuring the lived experience is at the forefront of the research process, we used latent class analysis to look at the characteristics of our sample and took it one step further by assessing these profiles over time.

Three distinct profiles were found using baseline indicator data, relative to the other profiles; for profile one (*N* = 99; lMlDhR), we found reports of the lowest microaggression, lowest discrimination, and the highest resilience experiences. In the second profile (*N* = 41; hMhDmR: high microaggression, high discrimination, moderate resilience), we found the highest microaggression experience, particularly related to sexual minority status followed by the highest discrimination experience and moderate resilience compared to the first and third profiles. And finally, in our third profile (*N* = 11; mMmDlR), we found that compared to the other profiles, individuals in this group were more likely to report moderate levels of microaggression and discrimination experience and report the lowest resilience experience. Individuals in profile 3 were more likely to report feeling microaggressions related to living with HIV and lower levels of resilience particularly in the area of social support. Previous studies have looked at the importance of social support with HIV-related health outcomes [16, 60]. Another striking finding in our study is how the profiles compared and changed over time (Fig. 2.). Results revealed that compared to profile one (least microaggression, least discrimination, and highest resilience (lMlDhR), the individuals in profile 2 (hMhDmR) displayed higher discrimination at the last time point and higher resilience at the second time point. Profile 3 displayed higher microaggression, higher resilience at the last time point, and higher discrimination at time point 2. Comparing profile 3 (mMmDlR) and profile 2, the former profile had lower microaggression. These results highlight that the changes found in mean factor scores seen in the overall sample were not observed for all individuals when looking at subgroups. This should not come as a surprise given that intersectional adversities (e.g., racism, sexism, HIV stigma) are external stressors that can happen at any time in the context of the oppression faced by BWLWH. Similarly, while resilience resources (e.g., self-efficacy, post-traumatic growth, social support) may improve over time, they may also decrease with repeated exposure to adversities and decreased social support due to losses. In general, it is important for researchers to consider this during intervention development.

## Strengths, Limitations, and Future Directions

There are several strengths of this study. Firstly, this study compared factor scores using longitudinal data. This is innovative and builds on pre-existing work [22] that used cross-sectional latent class analysis to examine adversity and resilience factors among BWLWH. In addition, the current findings include resilience resources over time on both individual and interpersonal levels. Second, this study illustrated details of a practical method to compare factor means of psychosocial domains in HIV research. The pre-condition of measurement invariance was met, and this study compared differences in the means over time of factor scores of psychosocial measurements by latent classes. Beyond the strengths, there are limitations worth noting. The conclusion of MI can be affected by the aspects of data being collected, the length of data collection, and the number of waves of data. The precision of results may have been impacted by a small amount of sample attrition and some decrease in measure response over time. However, since the invariance analyses were based on fit statistics and not statistical tests, the difference in precision should not have had an effect on those analyses. Estimated means of the factors may have been affected if persons not in the later times were different from those at baseline. Also, the COVID-19 pandemic onset and the Black Lives Matter protests may have impacted the variable means at later times (e.g., T3). For instance, microaggressions and discriminations may have been amplified as the U.S. navigated the murder of George Floyd, the murder of Breanna Taylor, and the Black Lives Matter protest. Similarly, intentional narratives on Black resistance and history around protests as well as BWLWH recognizing that they had applicable tools from navigating an HIV epidemic to apply to the COVID-19 pandemic may have boosted their reported resilience. However, the true period effects of the COVID-19 pandemic and Black Lives Matter protests remain unclear, and future work is needed to explore. Future studies with a larger number of participants, more waves of data, and additional analyses (e.g., growth mixture model) may be beneficial and continue to improve our understanding.

In summary, among BWLWH, this study examined (a) the measurement invariance of discrimination, microaggressions, and resilience and (b) the interplay of discrimination, microaggressions, and resilience across time utilizing latent class and repeated measure analysis. Latent factors were developed to capture discrimination, microaggressions, and resilience across three time points and latent class analysis identified three classes based on varying levels of discrimination, microaggressions, and resilience reported. Our findings indicated that (a) measurement invariance was partially met and (b) latent factor mean scores of microaggressions and discrimination decreased after 3 and 6 months and increased after 6 months for resilience and varied over time across the three latent classes identified. The subgroup with the lowest level of discrimination and microaggressions and the highest level of resilience reported at baseline experienced increases in resilience at months 3 and 6. Specifically, for those with the lowest level of discrimination and microaggression and the highest level of resilience at baseline, the resilience factor mean increased at a later time. This study also found that BWLWH who experienced the highest level of discrimination had the lowest level of resilience resources (individual and interpersonal). Findings establish a key prerequisite (measurement invariance) to examine relationships between discrimination, microaggressions, and resilience across time and suggest a negative link between discrimination and resilience, which echoes previous findings that microaggression has a negative impact on resilience [19].

## Supplementary Material

Supplementary Material 1

## Figures and Tables

**Table 1:** Sociodemographic characteristics among 151 BWLWH

	Frequency (/mean)	Percent (/SD)
Age (average)	52.89	10.45
Ethnicity		
Non-Hispanic	146	96.69
Hispanic	5	3.31
Education		
Eighth grade or lower	6	4.00
Some high school	48	32.00
High school graduate or GED	58	38.67
Some college	24	16.00
College graduate	9	6.00
Some graduate school	2	1.33
Graduate school degree	3	2.00
Sex orientation		
Heterosexual	124	85.52
Same gender loving (gay or lesbian)	4	2.76
Bisexual	12	8.28
Asexual	4	2.76
Income		
Less than $5000	36	27.27
$5000 through $11,999	61	46.21
$12,000 through $15,999	11	8.33
$16,000 through $24,999	11	8.33
$25,000 through $34,999	6	4.55
$35,000 through $49,999	5	3.79
$50,000 and greater	2	1.52
Housing		
Renting home or apartment	84	55.63
Living in home or apartment owned by you or else	19	12.58
Residential drug, alcohol, or other treatment facility	2	1.32
Publicly subsidized housing	26	17.22
A friend or relative’s home/apartment	12	7.95
Temporary/transitional housing	2	1.32
Homeless: sleeping in a shelter	5	3.31
Other	1	0.66
Employment		
Full-time work	13	8.78
Part-time work	14	9.46
Full-time or part-time in school	1	0.68
Neither in work nor in school	15	10.14
On disability	101	68.24
Other	4	2.70
Relationship status		
Married	18	12.16
Not married, but living with someone as if married	23	15.54
Non-cohabiting relationship (in a relationship but we do not live together)	13	8.78
Single	70	47.30
Divorced or separated	11	7.43
Loss of long-term partner/widowed	13	8.78

**Table 2:** Fit indices of invariance tests

	Configural invariance	Metric invariance		Scalar invariance		Strict invariance			
						Item variances		Factor variances	
		Initial	Final	Initial	Final	Initial	Final	Initial	Final
Akaike (AIC) *	21,755.9	21.843.6	21,758.4	22,023.3	21,764.4	22,023.3	21,775.0	21,828.7	21,782.5
Bayesian (BIC) *	22,202.5	22,247.9	22,171.7	22,358.2	22,153.6	22,358.2	22,122.0	22,157.6	22,117.4
Sample size–adjusted BIC*	21,734.1	21,823.8	21,738.2	22,006.9	21,745.4	22,006.9	21,758.0	21,812.6	21,766.1
RMSEA *	0.058	0.068	0.054	0.085	0.056	0.085	0.054	0.060	0.055
CFI *	0.907	0.865	0.914	0.773	0.906	0.773	0.910	0.886	0.904

*AIC* Akaike information criterion, *BIC* Bayesian information criterion, *RMSEA* root mean square error of approximation, *CIE* comparative fit index

**Table 3:** Coefficients of loadings of latent factors of microaggression, discrimination, and resilience across time

Factor loadings	Conligural invariance		Metric invariance				Scalar invariance				Strict invariance Final models			
			Initial model		Final model		Initial model		Final model		Item variance		Factor variance	
	Raw	SE	Raw	SE	Raw	SE	Raw	SE	Raw	SE	Raw	SE	Raw	SE
MICRO T1														
GRMS * T1	1	0	1	0	1	0	1	0	1	0	1	0	1	0
LGBT * T1	11.63	3.33	12.03	4.14	9.99	2.81	41.59	7.87	9.73	2.32	9.28	2.18	10.07	2.23
HIVM * T1	9.04	1.40	9.25	1.80	9.16	1.14	12.86	2.59	9.51	1.16	8.94	1.19	9.58	1.17
MICRO T2														
GRMS * T2	1	0	1	0	1	0	1	0	1	0	1	0	1	0
LGBT * T2	8.74	3.21	12.03	4.14	9.99	2.81	41.59	7.87	9.73	2.32	9.28	2.18	10.07	2.23
HIVM * T2	9.87	1.82	9.25	1.80	9.16	1.14	12.86	2.59	9.51	1.16	8.94	1.19	9.58	1.17
MICRO T3														
GRMS * T3	1	0	1	0	1	0	1	0	1	0	1	0	1	0
LGBT * T3	0.09	0.10	12.03	4.14	0.11	0.11	0.24	0.39	0.11	0.11	0.11	0.11	0.10	0.10
HIVM * T3	8.83	1.86	9.25	1.80	9.16	1.14	12.86	2.59	9.51	1.16	8.94	1.19	9.58	1.17
MACRO T1														
MDSR * T1	1	0	1	0	1	0	1	0	1	0	1	0	1	0
MDSH * T1	1.00	0.11	1.06	0.12	1.05	0.13	0.99	0.08	1.04	0.13	1.07	0.12	1.07	0.12
MDSGE * T1	0.78	0.13	0.75	0.16	0.73	0.17	0.73	0.12	0.72	0.17	0.67	0.16	0.67	0.16
MACRO T2														
MDSR * T2	1	0	1	0	1	0	1	0	1	0	1	0	1	0
MDSH * T2	1.35	0.27	1.06	0.12	1.05	0.13	0.99	0.08	1.04	0.13	1.07	0.12	1.07	0.12
MDSGE * T2	0.73	0.28	0.75	0.16	0.73	0.17	0.73	0.12	0.72	0.17	0.67	0.16	0.67	0.16
MACRO T3														
MDSR * T3	1	0	1	0	1	0	1	0	1	0	1	0	1	0
MDSH * T3	0.98	0.22	1.06	0.12	1.05	0.13	0.99	0.08	1.04	0.13	1.07	0.12	1.07	0.12
MDSGE * T3	0.67	0.32	0.75	0.16	0.73	0.17	0.73	0.12	0.72	0.17	0.67	0.16	0.67	0.16
RESIL T1														
CD-RISC-10 * T1	1	0	1	0	1	0	1	0	1	0	1	0	1	0
PGI * T1	2.71	0.39	2.19	0.37	2.24	0.35	1.79	0.35	2.11	0.33	2.04	0.36	2.04	0.36
MDSPS * T1	0.63	1.08	0.94	0.17	0.94	0.16	0.94	0.18	0.96	0.16	0.93	0.17	0.96	0.17
GSE * T1	1	0.55	0.45	0.05	0.86	0.36	0.79	0.22	0.84	0.36	0.79	0.25	0.83	0.28
RES1L T2														
CD-RISC-10* T2	1	0	1	0	1	0	1	0	1	0	1	0	1	0
PGI * T2	2.05	0.51	2.19	0.37	2.24	0.35	1.79	0.35	2.11	0.33	2.04	0.36	2.04	0.36
MDSPS * T1	1.23	1.2	0.94	0.17	0.94	0.16	0.94	0.18	0.96	0.16	0.93	0.17	0.96	0.17
GSE * T1	−0.22	0.58	0.45	0.05	−0.09	0.41	0	0.21	−0.08	0.41	— 0.04	0.27	−0.05	0.29
RESIL T3														
CD-RISC-10 * T3	1	0	1	0	1	0	1	0	1	0	1	0	1	0
PGI * T3	1.01	0.52	2.19	0.37	2.24	0.35	1.79	0.35	2.11	0.33	2.04	0.36	2.04	0.36
MDSPS * T3	1.02	0.35	0.94	0.17	0.94	0.16	0.94	0.18	0.96	0.16	0.93	0.17	0.96	0.17
GSE * T3	0.57	0.15	0.45	0.05	0.63	0.15	0.6	0.11	0.61	0.11	0.62	0.11	0.58	0.09

*SE.* standard error. *GRMS,* Gendered Racial Microaggressions Scale-Black Women; *LGBT-PCMS,* LGBT People of Color Microaggressions Scale. *HIVM,* Adapted HIV Microaggression Scale; *MDSR,* Race subscale of the Multiple Discrimination Scale. *MDSH,* HIV subscale of the Multiple Discrimination; *MDSGE,* Adapted Gender subscale from the Multiple Discrimination. *CD-RISC-10,* Connor-Davidson Resilience Scale-10 Item: *PGI,* Post-traumatic Growth Inventory. *MDSPSS,* Multidimensional Scale of Perceived Social Support. *GSE,* Generalized Self-Efficacy Scale

**Table 4:** Intercepts of latent factors of microaggression, discrimination, and resilience across time

Intercepts	Contigural invariance		Metric invariance				Scalar invariance				Strict invariance Final models			
			Initial model		Final model		Initial model		Final model		Item variance		Factor variancc	
	Raw	SE	Raw	SE	Raw	SE	Raw	SE	Raw	SE	Raw	SE	Raw	SE
GRMS T1	1.47	0.09	1.47	0.09	1.47	0.09	1.47	0.09	1.45	0.09	1.45	0.09	1.45	0.09
LGBT-PCMS T1	9.4	1.28	9.4	1.28	9.4	1.28	9.74	1.31	9.65	1.33	9.6	1.28	9.65	1.28
HIVM T1	16.77	0.78	16.77	0.78	16.77	0.78	14.97	0.74	16.49	0.79	16.46	0.77	16.51	0.78
MDSR T1	2.47	0.24	2.47	0.24	2.47	0.24	2.44	0.23	2.41	0.23	2.41	0.23	2.42	0.23
MDSH T1	1.67	0.2	1.67	0.2	1.67	0.2	1.64	0.2	1.64	0.2	1.64	0.2	1.64	0.2
MDSGE T1	1.17	0.19	1.17	0.19	1.17	0.19	1.16	0.19	1.18	0.19	1.18	0.19	1.19	0.19
GRMS T2	1	0.07	1	0.07	1	0.07	1.17	0.08	1.52	0.12	1.55	0.13	1.51	0.12
LGBT-PCMS T2	5.09	1.02	5.09	1.02	5.09	1.02	9.74	1.31	9.65	1.33	9.6	1.28	9.65	1.28
HIVM T2	12.04	0.74	12.02	0.74	12.03	0.74	14.97	0.74	16.49	0.79	16.46	0.77	16.51	0.78
MDSR T2	1.13	0.17	1.12	0.17	1.13	0.17	2.44	0.23	2.41	0.23	2.41	0.23	2.42	0.23
MDSH T2	0.8	0.18	0.78	0.17	0.78	0.17	2.19	0.29	2.29	0.34	2.34	0.33	2.36	0.33
MDSGE T2	0.4	0.11	0.4	0.11	0.4	0.11	1.5	0.29	1.5	0.35	1.44	0.34	1.44	0.34
GRMS T3	0.87	0.07	0.87	0.07	0.87	0.07	1.17	0.08	1.52	0.12	1.55	0.13	1.51	0.12
LGBT-PCMS T3	0.12	0.07	0.12	0.06	0.12	0.07	0.19	0.17	0.19	0.12	0.2	0.13	0.18	0.11
HIVM T3	9.86	0.73	9.71	0.73	9.85	0.73	14.97	0.74	16.49	0.79	16.46	0.77	16.51	0.78
MDSR T3	0.6	0.13	0.59	0.13	0.59	0.13	2.44	0.23	2.41	0.23	2.41	0.23	2.42	0.23
MDSH T3	0.47	0.12	0.46	0.12	0.47	0.12	2.19	0.29	2.29	0.34	2.34	0.33	2.36	0.33
MDSGE T3	0.31	0.1	0.3	0.1	0.31	0.1	1.5	0.29	1.5	0.35	1.44	0.34	1.44	0.34
CDRS T1	26.39	0.73	26.4	0.72	26.38	0.73	27.01	0.72	27.08	0.71	27.17	0.72	27.1	0.72
PGI T1	71.73	1.91	71.78	1.9	71.78	1.9	69.11	1.61	70.4	1.78	70.35	1.77	70.17	1.75
MDSPS T1	59.43	1.49	59.36	1.49	59.4	1.49	61.39	1.33	61.54	1.37	61.58	1.33	61.46	1.33
GSE T1	30.85	0.58	30.84	0.58	30.85	0.58	31.13	0.56	31.18	0.54	31.17	0.56	31.17	0.55
CD-RISC-10 T2	27.12	0.77	27.15	0.77	27.12	0.77	27.01	0.72	27.08	0.71	27.17	0.72	27.1	0.72
PGI T2	69.91	2.32	69.99	2.33	69.88	2.33	69.11	1.61	70.4	1.78	70.35	1.77	70.17	1.75
CD-RISC-10 T3	29.73	0.71	29.74	0.71	29.72	0.71	27.01	0.72	27.08	0.71	27.17	0.72	27.1	0.72
PGI T3	70.07	2.35	70.2	2.34	70.17	2.33	69.11	1.61	70.4	1.78	70.35	1.77	70.17	1.75
MDSPS T3	65.75	1.31	65.71	1.31	65.68	1.31	61.39	1.33	61.54	1.37	61.58	1.33	61.46	1.33
GSE T3	32.69	0.52	32.64	0.53	32.68	0.52	31.13	0.56	31.18	0.54	31.17	0.56	31.17	0.55

*GRMS,* Gendered Racial Microaggressions Scale-Black Women. *LGBT-PCMS,* LGBT People of Color Microaggressions Scale. *HIVM,* Adapted HIV Microaggression Scale. *MDSR,* Race subscale of the Multiple Discrimination Scale. *MDSH,* HIV subscale of the Multiple Discrimination. *MDSGE,* Adapted Gender subscale from the Multiple Discrimination. *CD-RISC-10,* Connor-Davidson Resilience Scale-10 Item. *PGI,* Post-traumatic Growth Inventory. *MDSPSS,* Multidimensional Scale of Perceived Social Support. *GSE,* Generalized Self-Efficacy Scale

**Table 5:** Variance and residual variance of latent factors of microaggression, discrimination, and resilience across time

Coefficients	Configural invariance		Metric invariance				Scalar invariance				Strict invariance Final models			
			Initial model		Final model		Initial model		Final model		Item variance		Factor variance	
	Raw	SE	Raw	SE	Raw	SE	Raw	SE	Raw	SE	Raw	SE	Raw	SE
Variances														
MICRO T1	0.63	0.16	0.50	0.19	0.65	0.15	0.14	0.06	0.63	0.15	0.73	0.17	0.49	0.10
MICRO T2	0.42	0.13	0.26	0.09	0.43	0.10	0.09	0.04	0.42	0.10	0.44	0.10	0.49	0.10
MICRO T3	0.36	0.10	0	0	0.35	0.08	0.07	0.05	0.34	0.08	0.33	0.08	0.49	0.10
MACRO T1	5.26	1.24	4.96	1.53	5.08	1.55	5.62	1.33	5.14	1.58	5.10	1.47	4.78	1.35
MACRO T2	1.72	0.50	1.99	0.60	2.04	0.60	2.34	0.74	2.07	0.60	2.49	0.71	2.75	0.80
MACRO T3	1.38	0.51	1.20	0.54	1.22	0.54	1.07	0.67	1.24	0.54	1.25	0.59	1.34	0.63
RESIL T1	44.62	10.53	49.67	10.9	48.86	10.4	49.98	12.03	50.39	10.7	51.77	11.13	44.02	8.40
RESIL T2	40.55	10.32	34.39	8.28	40.81	9.55	48.98	10.83	41.30	9.77	47.24	11.56	44.02	8.40
RESIL T3	39.55	10.14	36.68	7.83	32.91	8	35.76	7.43	34.30	7.74	34.38	7.59	44.02	8.40
Residual variance														
GRMS T1	0.52	0.11	0.57	0.12	0.51	0.10	0.76	0.09	0.51	0.10	0.36	0.07	0.39	0.06
LGBT-PCMS T1	169.13	31.99	176.93	34.18	175.48	32.74	0.61	0.36	177.53	31	147.07	23.81	145.18	22.93
HIVM T1	41.64	11.5	47.58	10.63	39.76	10.07	75.24	6.56	38.71	9.81	43.18	7.04	40.06	6.93
MDSR T1	2.93	0.55	3.10	0.59	3.07	0.58	2.05	0.50	3.06	0.59	2.88	0.78	2.90	0.78
MDSH T1	0.98	0.35	0.88	0.35	0.89	0.34	1.05	0.27	0.88	0.35	0.75	0.31	0.73	0.31
MDSGE T1	2.23	0.48	2.28	0.51	2.29	0.50	1.34	0.30	2.31	0.49	2.49	0.51	2.50	0.51
GRMS T2	0.36	0.11	0.43	0.11	0.35	0.10	0.76	0.09	0.35	0.09	0.36	0.07	0.39	0.06
LGBT-PCMS T2	112.78	27.86	108.28	31.61	111	28.35	0.61	0.36	111.63	29.03	147.07	23.81	145.18	22.93
HIVM T2	40.18	8.29	50.63	8.71	41.81	7.49	75.24	6.56	40.92	7.33	43.18	7.04	40.06	6.93
MDSR T2	2.58	1.27	2.68	1.25	2.57	1.26	2.05	0.50	2.61	1.31	2.88	0.78	2.90	0.78
MDSH T2	1.03	0.46	1.40	0.56	1.37	0.52	1.05	0.27	1.37	0.52	0.75	0.31	0.73	0.31
MDSGE T2	0.60	0.17	0.51	0.17	0.55	0.17	1.34	0.30	0.56	0.17	0.73	0.25	0.73	0.25
GRMS T3	0.26	0.08	0.58	0.07	0.26	0.06	0.76	0.09	0.26	0.06	0.36	0.07	0.39	0.06
LGBT-PCMS T3	0.61	0.36	1.08	0.75	0.61	0.36	0.61	0.36	0.61	0.36	0.62	0.37	0.62	0.37
HIVM T3	44.72	9.93	74.35	9.40	44.38	9.34	75.24	6.56	43.64	9.43	43.18	7.04	40.06	6.93
MDSR T3	0.96	0.41	1.09	0.44	1.03	0.43	2.05	0.50	1.03	0.43	0.95	0.40	0.96	0.40
MDSH T3	0.53	0.31	0.47	0.28	0.50	0.28	1.05	0.27	0.50	0.28	0.75	0.31	0.73	0.31
MDSGE T3	0.79	0.30	0.76	0.33	0.77	0.32	1.34	0.30	0.78	0.33	0.73	0.25	0.73	0.25
CD-RISC-10 T1	34.86	7.14	30.64	7.69	33.46	7.66	35.54	6.92	32.92	7.72	35.52	6.72	35.61	6.70
PGI T1	211.43	81.72	247.63	83.64	239.55	80.10	522.29	69.94	254.7	78.91	258	81.08	264.02	77.79
MDSPS T1	187.31	36.36	193.13	36.3	186.83	34.18	188.24	25.89	188.57	35	188.87	24.97	187.3	24.89
GSE T1	19.69	5.07	21.34	5.14	20.06	4.69	21.55	3.52	20.05	4.68	21.78	3.38	21.79	3.34
CD-RISC-10 T2	48.07	10.14	49.64	8.34	47.37	9.52	35.54	6.92	47.14	9.47	35.52	6.72	35.61	6.70
PGI T2	580.76	133.40	582.47	119.85	565.29	125.12	522.29	69.94	572.19	124.21	649.66	91.59	650.63	92.54
CD-RISC-10 T3	30.04	8.01	32.81	7	34.22	6.60	35.54	6.92	33.68	6.82	35.52	6.72	35.61	6.70
PGI T3	701.16	117.01	691.5	127.4	692.82	127.39	522.29	69.94	726.51	145	649.66	91.59	650.63	92.54
MDSPS T3	180.99	28.89	182.03	26.07	182.47	25.62	188.24	25.89	184.95	25.99	188.87	24.97	187.3	24.89
GSE T3	23.48	4.73	25.89	4.21	23.48	4.92	21.55	3.52	23.55	4.68	21.78	3.38	21.79	3.34

*SE,* standard error. *GRMS,* Gendered Racial Microaggressions Scale-Black Women; *LGBT-PCMS,* LGBT People of Color Microaggressions Scale; *HIVM,* Adapted HIV Microaggression Scale; *MDSR,* Race subscale of the Multiple Discrimination Scale; *MDSH,* HIV subscale of the Multiple Discrimination; *MDSGE,* Adapted Gender subscale from the Multiple Discrimination; *CD-RISC-10,* Connor-Davidson Resilience Scale-10 Item; *PGI,* Post-traumatic Growth Inventory; *MDSPSS,* Multidimensional Scale of Perceived Social Support; *GSE,* Generalized Self-Efticacy Scale

**Table 6:** Standardized coefficients for the final model

		Strict invariance Final models			
Standardized coefficients	Predictors	Estimate	Standard error	Z-stalistics	*P*-value
MICRO 1	GRMS T1	0.745	0.051	14.73	0
	LGBT-PCMS T1	0.505	0.067	7.519	0
	HIVM T1	0.727	0.054	13.38	0
MICR02	GRMS T2	0.745	0.051	14.73	0
	LGBT-PCMS T2	0.505	0.067	7.519	0
	HIVM T2	0.727	0.054	13.38	0
MICRO3	GRMS T3	0.745	0.051	14.73	0
	LGBT-PCMS T3	0.089	0.071	1.248	0.212
	HIVM T3	0.727	0.054	13.38	0
MACRO1	MDSR T1	0.789	0.065	12.22	0
	MDSH T1	0.939	0.029	32.489	0
	MDSGE T1	0.681	0.101	6.772	0
MACR02	MDSR T2	0.698	0.062	11.191	0
	MDSH T2	0.901	0.061	14.658	0
	MDSGE T2	0.794	0.052	15.375	0
MACRO3	MDSR T3	0.764	0.101	7.58	0
	MDSH T3	0.823	0.107	7.718	0
	MDSGE T3	0.673	0.118	5.716	0
RESIL1	CD-RISC-10 T1	0.744	0.058	12.854	0
	PGI T1	0.641	0.101	6.316	0
	MDSPS T1	0.349	0.035	9.962	0
	GSE T1	0.785	0.218	3.603	0
RESIL2	CD-RISC-10 T2	0.744	0.058	12.854	0
	PGI T2	0.469	0.062	7.551	0
	MDSPS T1	0.349	0.035	9.962	0
	GSE T1	−0.047	0.268	−0.177	0.859
RESIL3	CD-RISC-10 T3	0.744	0.058	12.854	0
	PGI T3	0.469	0.062	7.551	0
	MDSPS T3	0.42	0.059	7.119	0
	GSE T3	0.639	0.077	8.289	0

*GRMS*, Gendered Racial Microaggressions Scale-Black Women; *LGBT-PCMS,* LGBT People of Color Microaggressions Scale; *HIVM,* Adapted HIV Microaggression Scale; *MDSR,* Race subscale of the Multiple Discrimination Scale; *MDSH,* HIV subscale of the Multiple Discrimination; *MDSGE,* Adapted Gender subscale from the Multiple Discrimination; *CD-RISC-10,* Connor-Davidson Resilience Scale-10 Item; *PGI,* Post-traumatic Growth Inventory; *MDSPSS,* Multidimensional Scale of Perceived Social Support; *GSE,* Generalized Self-Efficacy Scale

**Table 7:** Means of latent factors between the three latent classes over time

Factor means (SD)	Highest resilient and lowest discrimination and microaggression (Class 1) *N=*99	Highest discrimination and microaggression (Class 2) *N* = 41	Lowest resilient (Class 3) *N* = 11	Overall test Chi-square	Overall test *P*-value
MICRO T1	−0.432 (0.037)	1.121 (0.148)	0.751 (0.071)	280.145*	0.000
MICRO T2	−0.73 (0.045)	0.219 (0.164)	−0.114 (0.091)	58.276*	0.000
MICRO T3	−0.851 (0.04)	−0.191 (0.192)	−0.348 (0.103)	28.193*	0.000
DISCRI T1	− 1.044 (0.094)	5.872 (0.646)	0.992 (0.233)	164.496*	0.000
DISCRI T2	−1.936 (0.081)	0.868 (0.715)	−0.997 (0.284)	23.683*	0.000
DISCRI T3	−2.056 (0.05)	−0.841 (0.481)	−1.303 (0.229)	15.549*	0.000
RESIL T1	1.482 (0.615)	−2.339 (1.699)	−4.27 (0.907)	26.909*	0.000
RESIL T2	1.463 (0.581)	1.444 (1.28)	−4.34 (0.804)	33.974*	0.000
RESIL T3	3.61 (0.54)	4.269 (1.489)	−0.157 (0.811)	15.287*	0.001

* Indicated significant at .05 statistical level

## Data Availability

The study is ongoing therefore data is unavailable.
