# Supplementary material for: Testing for Measurement Invariance (MI): Do the Structures of Microaggression, Discrimination, and Resilience Among Black Women Living with HIV Remain the Same Across Time?
Source: J Racial Ethn Health Disparities. 2024 Aug 5;12(4):2752–70. doi: 10.1007/s40615-024-02087-w (PMC12184700; doi:10.1007/s40615-024-02087-w)
Supplement: Supplementary file 1 — Supplementary file1 (DOCX 28.3 KB) [file 40615_2024_2087_MOESM1_ESM.docx]

**Supplemental Tables**

**Table 8**

Factor means of the final model.

|  | Estimate | Standard Error | Z-statistics | *P*-value |
| --- | --- | --- | --- | --- |
| MICRO* T2 | -0.494 | 0.101 | -4.886 | 0 |
| MICRO* T3 | -0.662 | 0.113 | -5.831 | 0 |
| MACRO* T2 | -1.481 | 0.207 | -7.146 | 0 |
| MACRO* T3 | -1.758 | 0.226 | -7.789 | 0 |
| RESIL* T2 | 0.144 | 0.609 | 0.236 | 0.814 |
| RESIL* T3 | 2.815 | 0.744 | 3.784 | 0 |

MICRO: latent factor of microaggression; MACRO: latent factor of discrimination; RESIL: latent factor of resilience.

**Table 9**

Summary of LCA Model fit from 2-class solution to 5-class solution.

| Number of Latent Classes | Sample sizes in each class | Entropy* | AIC* | BIC* | Sample-Adjusted BIC* |
| --- | --- | --- | --- | --- | --- |
| 2 | 28  123 | 0.944 | 9360.994 | 9454.530 | 9356.418 |
| 3 | 99  41  11 | 0.920 | 9183.843 | 9310.569 | 9177.643 |
| 4 | 40  62  38  11 | 0.860 | 9111.320 | 9271.236 | 9103.496 |
| 5 | 5  46  33  56  11 | 0.875 | 9052.873 | 9245.979 | 9043.425 |

AIC: Akaike Information Criterion; BIC: Bayesian Information Criterion; RMSEA: Root mean square error of approximation; CIF: Comparative fit index.

**Table 10**

The means (SD) of observed items at baseline for three latent classes.

| Latent Class Indicators (Organized by CFA Latent Factors) | Baseline  Items | Highest resilient and lowest discrimination and microaggression  (Class 1)  N=99 | Highest discrimination and microaggression  (Class 2)  N=41 | Lowest resilient  (Class 3)  N=11 |
| --- | --- | --- | --- | --- |
| Microaggression | GRMS | 0.897 (.08) | 2.504 (.27) | 2.526 (.09) |
|  | LGBT-PCMS | 3.888 (.84) | 30.499 (7.29) | 16.669 (28.34) |
|  | HIVM | 12.115 (.95) | 24.567 (1.89) | 25.458 (5.22) |
| Discrimination | MDSRACE | 1.109 (.20) | 9.208 (.81) | 3.818 (.52) |
|  | MDSHIV | 0.553 (.15) | 8.518 (.77) | 2.431 (.37) |
|  | MDSGENDER | 0.411 (.10) | 7.562 (1.11) | 1.26 (.50) |
| Resilience | CD-RISC-10 | 27.993 (.98) | 25.834 (1.78) | 23.532 (9.07) |
|  | PGI | 76.679 (2.61) | 69.022 (5.03) | 62.393 (73.57) |
|  | MDSPS | 64.406 (1.95) | 60.895 (4.70) | 48.265 (34.77) |
|  | GSE | 32.033 (0.75) | 29.602 (2.40) | 28.398 (7.37) |

GRMS: Gendered Racial Microaggressions Scale-Black Women; LGBT-PCMS: LGBT People of Color Microaggressions Scale; HIVM: Adapted HIV Microaggression Scale; MDSR: Race subscale of the Multiple Discrimination Scale; MDSH: HIV subscale of the Multiple Discrimination; MDSGE: Adapted Gender subscale from the Multiple Discrimination; CD-RISC-10: Connor-Davidson Resilience Scale-10 Item; PGI: Post-traumatic Growth Inventory; MDSPSS: Multidimensional Scale of Perceived Social Support; GSE: Generalized Self-Efficacy Scale

**Table 11**

Comparisons of Means of latent factors between the three latent classes.

| Chi-Square | Class 2  Vs.  Class 1 | Class 3  Vs.  Class 1 | Class 3  Vs.  class 2 |
| --- | --- | --- | --- |
| MICRO T1 | 97.025 * | 205.439 * | 6.574 * |
| MICRO T2 | 31.283 * | 25.880 * | 2.172 |
| MICRO T3 | 11.230 * | 15.863 * | 0.367 |
| DISCRI T1 | 80.810 * | 51.040 * | 37.377 * |
| DISCRI T2 | 10.086 * | 8.629 * | 3.654 |
| DISCRI T3 | 6.005 * | 10.529 * | 0.865 |
| RESIL T1 | 4.783 * | 24.097 * | 1.180 |
| RESIL T2 | 0.008 | 29.641 * | 15.061 * |
| RESIL T3 | 0.147 | 13.042 * | 6.932 * |

* Indicated significant at .05 statistical level

**Table 12**

Factor Correlations

|  | **MICRO1** | **MICRO2** | **MICRO3** | **MACRO1** | **MACRO2** | **MACRO3** | **RESIL1** | **RESIL2** | **RESIL3** |
| --- | --- | --- | --- | --- | --- | --- | --- | --- | --- |
| **MICRO1** | 1 | 0.604 | 0.458 | 0.629 | 0.330 | 0.323 | -0.302 | -0.296 | -0.240 |
| **MICRO2** |  | 1 | 0.813 | 0.454 | 0.602 | 0.443 | -0.128 | -0.284 | -0.485 |
| **MICRO3** |  |  | 1 | 0.352 | 0.707 | 0.666 | -0.004 | -0.276 | -0.383 |
| **MACRO1** |  |  |  | 1 | 0.628 | 0.430 | -0.201 | -0.033 | 0.013 |
| **MACRO2** |  |  |  |  | 1 | 0.845 | -0.109 | -0.195 | -0.285 |
| **MACRO3** |  |  |  |  |  | 1 | -0.020 | -0.238 | -0.064 |
| **RESIL1** |  |  |  |  |  |  | 1 | 0.764 | 0.528 |
| **RESIL2** |  |  |  |  |  |  |  | 1 | 0.678 |
| **RESIL3** |  |  |  |  |  |  |  |  | 1 |

**Table 13**

Sample size of response to measures overtime

| **Measure** |  |  |  |
| --- | --- | --- | --- |
|  | ***Time 1*** | ***Time 2*** | ***Time 3*** |
| **GRMS** | 144 | 140 | 135 |
| **LGBT** | 151 | 146 | 135 |
| **HIVM** | 151 | 144 | 133 |
| **MDSR** | 148 | 138 | 134 |
| **MDSH** | 147 | 141 | 134 |
| **MDSGE** | 151 | 145 | 135 |
| **CDRS** | 148 | 140 | 135 |
| **MDSPS** | 147 | 140 | 128 |
| **GSE** | 150 | 143 | 128 |
| **PGI** | 148 | 136 | 135 |

GRMS: Gendered Racial Microaggressions Scale-Black Women; LGBT-PCMS: LGBT People of Color Microaggressions Scale; HIVM: Adapted HIV Microaggression Scale; MDSR: Race subscale of the Multiple Discrimination Scale; MDSH: HIV subscale of the Multiple Discrimination; MDSGE: Adapted Gender subscale from the Multiple Discrimination; CD-RISC-10: Connor-Davidson Resilience Scale-10 Item; PGI: Post-traumatic Growth Inventory; MDSPSS: Multidimensional Scale of Perceived Social Support; GSE: Generalized Self-Efficacy Scale
